# Interpreting weights of multimodal machine learning models—problems and pitfalls

**DOI:** 10.1038/s41386-021-01030-5

**Published:** 2021-05-20

**Authors:** Nils Ralf Winter, Janik Goltermann, Udo Dannlowski, Tim Hahn

**Affiliations:** Institute for Translational Psychiatry, University of Münster, Münster, Germany

**Keywords:** Neuroscience, Biomarkers

Using machine learning, Price et al. [1] aim to uncover “*if and how brain structure distinguished young adults with and without a history of maltreatment*”. From their analysis and the respective importance of variables (model weights), they conclude that subjects with a history of maltreatment display specific alterations in cortical surface area and thickness of multiple brain regions.

Crucially – in addition to the cortical and subcortical brain variables – the authors include “non-brain” variables, namely socioeconomic status, cognitive functioning, psychopathology, as well as age, gender, and the scanning site as features in their machine learning model. We argue that their results (1) do not provide any information regarding the unique association of brain structure and childhood maltreatment and (2) are likely due to generally well-known associations between clinical variables and childhood maltreatment. Notably, a study referenced by Price et al. used a similar statistical approach and thus suffers from the same issues outlined below [2].

Specifically, once these “non-brain” variables are added to the predictive model, the performance of a model based on brain data alone simply cannot be determined anymore. The reason for this arises from the nature of multivariate models in general: As all weights are jointly estimated, every additional variable may change all other weights already in the model. Thus, the authors’ analysis is uninformative with regard to the unique contribution of brain variables whenever a single “non-brain” variable is present in the model. Drawing a subset of variables multiple times as done by the authors does not change this fact if a single non-brain variable remains in the drawn feature set. This so-called Rashomon effect is well-known in statistics [3].

Secondly, the authors interpret multivariate weights as if they were univariate associations. Importantly, however, a large weight does not imply a strong association with maltreatment in this context. For example, even a variable without any association may receive a large weight if it explains error variance in other variables completely independent of maltreatment (cf. Suppression Effect in Capraro and Capraro [4]). Thus, considering importance maps cannot remedy the problem outlined above.

Third, the relatively good model performance found by the authors is most likely based on the known association between clinical variables and childhood maltreatment. As it can be safely assumed that psychopathology and socioeconomic status are highly associated with a history of childhood maltreatment [5], model performance is likely driven by these variables. The authors even provide evidence towards this point reporting that their maltreatment group had a significantly lower socioeconomic status and higher psychopathology. An analysis of model weights cannot counter this argument due to the Rashomon and the Suppression Effects outlined above.

Finally, the authors also include scanner site, age, and gender in their model, variables classically controlled for in statistical inference. In this study, however, they are explicitly used to predict maltreatment experience. As at least scanner site and age receive non-zero weights, we can be sure that model performance is at least in part driven by these confounding variables.

Despite these fundamental problems, a remedy is simple: As suggested for example by Yarkoni and Westfall [6], one can estimate the relative contribution of specific variables by comparing a ‘full’ model containing all available variables with a partial model that only contains a subset of features. Thus, the authors need to verify their claims by showing that a model containing *brain variables only* still displays similar performance. As this does not control for the effect of confounding variables, consistent results would need to be shown across scanner site, age, and gender as well.

## Funding and disclosure

Open Access funding enabled and organized by Projekt DEAL. This work was supported by grants from the Interdisciplinary Center for Clinical Research (IZKF) of the medical faculty of Münster (grant Dan3/012/17 to UD) and the German Research Foundation (DFG grants HA7070/2-2, HA7070/3, HA7070/4 to TH). The authors declare no competing interests.
